# The role of peer support in coping and adjustment to dialysis and transplantation: Study protocol

**DOI:** 10.1371/journal.pone.0318124

**Published:** 2025-02-10

**Authors:** Anna Winterbottom, Eleri Wood, Andrew Mooney, Keith Bucknall, Hilary L. Bekker

**Affiliations:** 1 Adult Renal Services, Lincoln Wing, St James University Hospital, Leeds, United Kingdom; 2 Leeds Institute of Health Sciences, School of Medicine, University of Leeds, Leeds, United Kingdom; 3 Kings College Hospital, London, United Kingdom; 4 Patient Representative, Devon, United Kingdom; PLOS: Public Library of Science, UNITED KINGDOM OF GREAT BRITAIN AND NORTHERN IRELAND

## Abstract

**Introduction:**

People with kidney failure receiving dialysis or kidney transplantation find it difficult to adapt to treatment related routines and restrictions, and feel frustrated when their expectations aren’t matched by their lived experience. Health professionals provide information to help people prepare for kidney treatments, but it may be that ‘peer supporters’ - people who live with kidney disease - can provide more easily understood and relevant information. This study will explore how learning from peer supporters might improve the experience of treatment, after dialysis initiation and post-transplantation, by helping them to better understand what to expect from treatments.

**Methods:**

Two mixed methods studies including in-depth interviews and questionnaires. In each study, participants will be recruited at two timepoints, before commencing dialysis or transplantation, and 6 months later. Questionnaires and interviews will explore expectations and the lived experience of treatment, and if peer support impacts on adjustment and coping with treatment. Participants will be recruited from two large teaching hospitals in the North and South of England, where one has access to a formal kidney peer support program.

**Discussion:**

Delivering peer support in kidney units is increasingly popular, yet provision is inconsistent and generally low quality. Providing an evidence base for it’s use will help guide the optimal development of peer support programmes and efficient allocation of peer resources. A report will be produced to summarise the our findings, which will help kidney units better help people with kidney failure prepare for kidney treatments.

## Introduction

Kidney replacement therapies (KRT) (haemodialysis, peritoneal dialysis and transplantation), are intensive and life changing, requiring multiple adaptations to a person’s lifestyle [1–3]. Some people successfully adjust to treatments; others experience poor psychological outcomes. Many people undergoing dialysis report experiencing emotional distress, fear, anxiety, depression, loss, uncertainty, regret, guilt, and find the treatment burdensome [4–9]. Kidney transplant recipients may also experience fear and anxiety, difficulties adjusting to continued treatment burden, frustration, and disillusionment when post-transplant recovery and quality of life do not match expectations [10–12].

In the United Kingdom (UK) people approaching the need for KRT receive specialist kidney care including the provision of information and support by health professionals to help them make optimal decisions about kidney replacement therapies [13–15]. However, people report reluctance to accept the need for treatment, perceive a lack of choice, hold unrealistic expectations about prognosis and/or quality-of-life, and desire more psychosocial information [16–19]. It is suggested that regret experienced after dialysis initiation may be due to a mis-match between people’s expectations and subsequent experience [4,9,19,20].

Peer support is an innovative, policy-advocated method of providing informational, emotional and appraisal support [21]. Peer support involves people with kidney disease gaining an understanding from others with the same illness about their lived illness experience [22,23]. It may, when provided as an adjunct to education provided by health professionals, help people develop more realistic expectations of kidney replacement therapies and thus adjust better to, and be more satisfied with treatment [9,18,19].

Peer support is valued by people with kidney disease, providing encouragement, empathy, confidence, reassurance, and hope [24–26]. It uniquely utilizes layman’s terms to communicate health information, helping people understand the patient perspective of KRT and psychosocial consequences that may not be appreciated or conveyed by healthcare practitioners [24]. However, peer support provision across the UK is not routinely offered through kidney services; only 25% of units provide ‘formal’ support (governed service provided by trained peers).

Little is known about mechanisms that make peer support ‘good’ or successful [23]. It is suggested, but poorly evidenced, that formal support from trained peers is better than informal support received from untrained peers (encountered incidentally in waiting rooms or social media) because trained peers are able to build rapport and less likely to present exaggerated, unbalanced, scary, information [27,28]. Also unknown is how the similarity or differences between supporter and peer support recipient (on dimensions such as age, gender, and ethnicity) influence its outcomes [24,27]. It is hypothesised that the greater the similarity, the more empathy, trust, and role-modelling can occur, and therefore the greater the benefits. This may be particularly relevant for minority groups. People with kidney failure desire culturally and linguistically appropriate treatment information [29].

Peer support is uniquely placed to provide culturally relevant information by targeting it towards specific ethnic minority groups, and by matching supporters and recipients from local backgrounds [30]. A trial of peer support for people receiving haemodialysis showed that it preferentially benefitted those from ethnic minority groups [31]. Low health literacy is common in people with kidney failure and is associated with poor knowledge about kidney disease, self-management behaviours and health‐related quality-of-life [32–35]. Peer support can also help improve people’s understanding of health information by providing information in an accessible, patient centred and relatable format [36].

Whilst developing our study, we conducted a focus group as part of our patient and public involvement (PPI) activities, to learn how well people felt they were prepared for KRT. Participants identified that a) people with kidney failure who are unwell when they start treatment have little time to contemplate what dialysis would entail and prepare for changes, b) people with kidney failure who are able to talk with other people whilst having treatment, are more likely to cope better, c) some people struggle to cope with the adjustments required to fit dialysis into daily life, and d) some people are fearful about the impact that treatment could have on family relationships. The PPI participants also discussed the associated ‘treatment burden’ of dialysis and felt that this aspect of treatment was not adequately addressed in current practice. For example, they would have valued more support on specific topics such as loss of libido and body image, to help prepare them for the lived experience of treatment. These findings support the theoretical basis for our research, namely that standard care insufficiently prepares people for the lived reality of life on KRT and that peer support may be an acceptable and successful intervention to make dialysis and transplant more tolerable and easy to live with.

Understanding the utility of peer support is important when assessing its impact, and the needs of services to integrate within their care pathway. International quality standards supporting healthcare decision making suggest whilst ‘patient narratives’ may improve health literacy, provide comfort and prepare people for treatment, they may bias people's decision making when they are deliberating between two or more treatment options [37,38]. Better understanding of the mechanisms of successful peer support will facilitate optimal development of peer programmes and allocation of peer resources.

### Aim

This research will identify active ingredients underpinning how peer support helps people adjust to kidney replacement therapies in order to design the most effective and efficient peer support programs.

### Objectives

1. Develop an in-depth understanding of the mechanisms and impact of standard care, formal and informal peer support, by interviewing people who have received different amounts and types of peer support, pre and post dialysis or transplantation.
2. Use survey methods to explore the impact of receiving peer support on people living with kidney disease experience, psychosocial and decision quality measures at two timepoints.
3. Produce a report of our findings to help refine current and future peer support programs.

## Materials and methods

### Design

A mixed methods approach including two studies to address research objectives 1&2. This approach allows investigation from two non-competing perspectives, an in-depth qualitative analysis of people living with kidney disease lived experiences, and a broader quantitative understanding of the topic, with each methodological approach addressing the design limitations inherent with the other [39]. The Good Reporting of A Mixed Methods Study (GRAMMS) guideline was followed [40].

### Patient and public involvement group

A patient and public involvement (PPI) group has been convened and will provide input to all stages of the project including, developing the interview schedule, selecting appropriate survey measures, dissemination activities, and report writing. An individual with dialysis and transplant experience is a co-applicant and will participate in steering group meetings and provide feedback to the wider PPI group.

### Setting

Recruitment for both studies will take place at Leeds Renal Unit which has ~ 400 advanced kidney care patients and King’s College Hospital, London which has ~550 advanced kidney care patients. These large inner-city hospitals include people with kidney disease from diverse social, religious, and cultural backgrounds. King’s College Hospital kidney unit has had an active, formal peer support service since 2006; Leeds does not; therefore, we will be recruiting from populations with different experiences of peer support.

### Materials

Study materials include consent forms, patient information sheets, interview schedule (study 1), questionnaire (study 2). The interview schedule and questionnaire will be developed by the research team in consultation with a patient and public involvement (PPI) team, and guided by the research aim, previous research examining patients’ expectations and experiences of kidney disease and its treatments.

### Ethics and research governance approvals

Local Research Ethics and Health Research Authority approval was granted by Health and Care Research Wales on 6th March, 2024 (IRAS project ID: 330749).

### Study 1 – in-depth interviews with people with kidney failure

We will develop a detailed understanding of people’s pre-treatment expectations of, and goals of care; the lived experience of treatment after commencing dialysis/post-transplantation; differences between the two; and how standard care and peer support of different types might influence both expectations and experience of treatment.

#### Sample size.

There is no formal analysis to estimate sample size in qualitative methods. As a guide, using our prior experience of interviewing this population, we estimate that approximately 25–30 people with kidney failure will be a reasonable sample size to generate sufficient data for these research questions. We will interview the same people at two different points of the patient pathway: Time 1 - pre-treatment to ascertain views around expectations and goals of care (T1), and at Time 2 - after commencing dialysis/post-transplantation about lived experience and treatment burden (T2), . From  our previous experience we know that interviewing people with kidney failure, with its associated high mortality rate, means that we may not be able to follow up everyone at Time 2. In this instance, findings recorded at T1 would still be used in the analysis, and if neccessary we will recruit additional people at Time 2 only. Our experiences of recruiting/interviewing at two timepoints will be documented in the final report. Recruitment will be discontinued when saturation is reached, and the author judges that no more new themes are being generated from the data [41].

At Time 1, adults with chronic kidney disease stages 4&5 (referred to herein as 'kidney failure') will be eligible to participate if they meet one of the following categories.

- Attending an Advanced Kidney Care Clinic and contemplating KRTs,

For those recruited at Time 2 only:

- Receiving haemodialysis or peritoneal dialysis – up to 6 months after commencement,
- Up to 6 months post-transplantation including people with a working transplant and those with graft failure,
- Who have received more than one KRT.

Purposive sampling [42] will ensure participants are recruited into three groups of roughly the same size based on experience of peer support – none, informal and formal. At recruitment, a screening question i.e., whether or not they have talked to anyone who has lived with KRT, will identify people who have received none or informal peer support. Using medical records, we will identify people (King’s College Hospital) who have documented evidence of receiving formal peer support. There is no upper age limit for participation. Participants must be able to take written, informed consent and be cognitively capable of taking part in an interview.

#### Recruitment.

The research nurse will work with kidney care staff supporting people at different stages of the pathway i.e., pre-treatment – advanced kidney care clinic clinicians, and post-treatment – dialysis and transplant clinicians, to identify people meeting the eligibility criteria stated above. Our extensive experience of recruiting from kidney services suggests a flexible and where possible, personal approach will ensure a sufficient number of participants are recruited. The research nurse will approach people attending clinics (advanced kidney care, post-transplantation, and PD) or haemodialysis sessions and discuss the study, hand out a patient information sheet and provide the opportunity for study related questions. With permission, those people we have approached will be contacted by telephone a week later about their decision to participate. We will write to people who are identified by staff, but who we are unable to access via an outpatient clinic or on the ward and send an introductory letter and patient information sheet in the post and ask them to return a reply slip with their contact details, if they are willing to take part.

**Recruitment of people from ethnic minority backgrounds:** We recognise that people taking part in kidney research studies are more likely to be more (health) literate, ‘white’ and from higher economic backgrounds. To help mitigate against this, we will undertake several steps. Ensuring smaller samples, particularly those in qualitative studies, are stratified on a number of criteria can prove challenging, however we will be mindful of, and guided by, local Trust and national databases e.g., renal registry, to improve the representativeness of our sample in terms of gender, age, ethnicity, and socio-economic status defined by postcode. The research nurse will offer to read through patient information and support survey completion for people who have difficulty reading, i.e., those with low literacy, eyesight problems, cannot read English. Learning from recent, successful research focussing on people with kidney disease from ethnic minority backgrounds [43], we will work with cultural improvement officers, interpreters, family members and members of the kidney team with similar backgrounds, to help boost recruitment of people from minority ethnic groups. Where necessary we will translate patient facing materials.

#### Procedure.

Pre-treatment interviews (T1) will include open-ended questions to understand people’s experience of adjusting to a diagnosis and/or treatment of kidney disease, expectations of KRT and goals of care, and experiences and impact of support from peers. Interview questions aimed at people on dialysis/post-transplant (T2) will explore how expectations of treatment match people’s lived experience, associated treatment burden, and any further experiences and impact of support from peers. Before commencing the interview, participants will have a further opportunity to ask study related questions and provide written consent to take part. Participants will be given the opportunity to have a relative or nurse present at interview. Permission will be sought from those taking part at T1, to be contacted 6 months later if they meet criteria for T2 interview i.e. have commenced KRT. Interviews will approximately 60 minutes and will be organised at participant’s convenience, either in home, at hospital, on the telephone, or using an online platform such as Microsoft Teams or Zoom. We will thank participants for their contribution to the research process by providing a £15 voucher.

#### Transcription, data coding, and analysis.

Audio recordings will be transcribed verbatim using standard protocols. Thematic analysis, taking account of the individuals narrative, will be used to analyse interview data [44] with the support of NVivo (Version 20.1.6) to organise the analysis and allow sharing amongst team members. Analysis will be conducted using a critical realist approach, whereby it is acknowledged that an external reality exists that is knowable and that people’s experiences are subjective. An initial coding frame will be generated which will be refined as analysis of individuals accounts, and emergent codes are generated. Each interview will be coded using a mixed deductive and inductive coding frame; 10% of the interviews will be coded by AW and a PPI member to maximise validity and robustness. Where discrepancy exists, the coders will reach consensus by referring to a third member of the research team. A thematic map will be generated using the method of constant comparison to illustrate the relationships between and within themes with input from AW.

### Study 2 – patient outcome survey comparing experiences standard care and peer support

Adopting a survey will allow a broad exploration of the impact on patient outcomes of receiving standard care compared to peer support. Using questionnaires, we will measure patient experience and psychological measures of coping and adjustment, treatment experience and satisfaction with peer support.

#### Sample.

As with study 1, we will recruit the same individuals at two points of the patient pathway: Time 1 - pre-treatment to ascertain views around expectations and goals of care (T1), and Time 2 - after commencing dialysis/post-transplantation about lived experience and treatment burden (T2), .The sample will be determined by the same eligibility criteria (clinical characteristics) and stratified (experience of peer support) as outlined for Study 1.

Our sample size calculation is based on a population of 950 (drawn from advanced kidney care, patients at both sites – as outlined in the ‘setting’ section) allowing for a 5% margin of error with 90% confidence, suggests 212 patients will be sufficient [45]. If necessary we will seek ethical approval to contact people with kidney failure via national charities and kidney patient organisations to reach the required sample size. 

#### Recruitment.

Participants will be identified using the methods detailed for Study 1. Where recruitment is face-to-face, surveys will be provided for people to complete at their own convenience and return in a stamped addressed envelope. Those eligible to take part but who cannot be approached directly will receive a covering letter, questionnaire and stamped addressed envelope via the postal service. Permission will be sought from those taking part at T1, to be contacted approximately 4-6 months later to complete a further survey. We will telephone participants and remind them to complete the questionnaire two weeks after initial contact . Interview and survey data collection will overlap to ensure project milestones are met. Participants eligible to participate in both studies will be invited into either study at the discretion of the research nurse to ensure a representative and diverse sample.

#### Materials.

Questionnaires were developed in consultation with the PPI advisory group to ensure their acceptability and relevance. They include previously established and where possible, validated measures to assess characteristics of the patient sample [demographics, patient history, peer support experience and satisfaction, physical symptoms, patient experience and psychological measures of coping and adjustment [goals and expectations of treatment and care, quality-of-life [46,47] treatment burden [48], To minimise questionnaire fatigue, we employ short-item questionnaires wherever possible. For example, the SURE measure assessing decisional conflict is 4 items.

#### Procedure.

Participants will be asked to complete a series of closed– and open-ended questions in the form of a questionnaire booklet. Survey completion will take place at a time and place convenient to the participant and take a maximum of 30 minutes. We will thank participants by giving them a £15 voucher for completing the survey. Participants will be required to return the survey using the postal service and a stamped addressed envelope provided by the research team.

#### Data analysis.

Descriptive statistics will summarize the sample characteristics. Multivariate analyses will look at differences in measures between groups by use of peer support/standard care. Repeated measures analyses will examine differences in experiences over time. Data will be managed using SPSS (Version 27).

### Objective 3 – study report

A study summary report will be produced to summarise our findings and identify the active ingredients of successful peer support. The findings will be disseminated at conferences, press releases and via scientific publication as agreed by the steering group and PPI team. Dissemination to wider patient charities and networks will be facilitated by PPI representatives. Findings will inform the work of the UK peer support working group, which one steering group member chairs.

## Discussion

Providing peer support in kidney units is increasingly popular, yet provision is inconsistent and generally low quality. At present, little is known about its utility in making dialysis and transplants more tolerable and easier to live with. Providing an evidence base for the use of peer support will a) provide the impetus to move the provision of peer support up the agenda with renal clinicians and commissioners, improve on the delivery of personalised care, and patient experience of care, and b) help guide the optimal development of peer support programmes and efficient allocation of peer resources as we see the development of regional networks for this popular quality improvement initiative [49].

## Supporting information

S1 File PS_questionnaire v.5 11112024_T1. Pre-treatment questionnaire, Time 1.
(DOCX)

S2 File PS_questionnaire v.5 11112024_T2. Post-treatment questionnaire, Time 2.
(DOCX)

S3 File Peer support_interview schedule Time 1 v.2 27.02.2024. Pre-treatment interview schedule, Time 1.
(DOCX)

S4 File Peer support_interview schedule Time 2 v.2 27.02.2024. Pre-treatment interview schedule, Time 2.
(DOCX)
