# Supplementary material for: The role of peer support in coping and adjustment to dialysis and transplantation: Study protocol
Source: PLoS One. 2025 Feb 10;20(2):e0318124. doi: 10.1371/journal.pone.0318124 (PMC11809911; doi:10.1371/journal.pone.0318124)
Supplement: S2 File — Post-treatment questionnaire, Time 2. (DOCX) [file pone.0318124.s002.docx]

*Office use only*

Study ID…………………

Time 1 2

**Peer Support Study: Questionnaire 2**

Thank you for agreeing to take part in this study on ***the role of peer support in helping people cope and adjust to dialysis and transplantation.***

Approximately 6 months ago, you completed a questionnaire for this study, and we are contacting you again to ask you to complete a second and final questionnaire.

Your views and experiences will be used by our study team to understand how kidney services can better support people with chronic kidney disease, and their peers.

There are 5 sections in the questionnaire, and it takes about 30 minutes to complete. Some people complete the questionnaire in one go, and others take a rest between sections. Your answers are anonymised, meaning that only the researcher can link your name with your answers. Your views and ideas will not be identifiable when the research is summarised or presented. Information you provide during the survey is confidential.

A patient information sheet, giving more details about the study and the research team is included with this questionnaire.

**What is Peer Support?**

People use the term peer support in different ways. Our study team describes peer support as:

Peer support is when people with similar long-term health conditions or health experiences come together to support each other to cope with their illness.

Peer support can happen formally when organised by a health service or charity, and informally when talking with a relative or another patient or family member. It can be part of a one-to-one conversation or as part of a group, in face-to-face or on-line communications. Peer support can happen when you are at home, at hospital, in community settings or travelling.

We are interested in people’s different experiences of peer support, including people who *haven’t experienced* any at all. *Please continue to complete this form even if you have no experience of peer support.*

**Section 1: Support from others**

This section asks you about your experience of talking to other people with kidney disease about your illness and treatment. *Please continue to complete this form even if you have no experience of talking with other people.*

| 1. **Have you spoken to anyone with kidney disease about your illness and/or treatment options?** *(circle your answer)*   YES NO | | | | |  |
| --- | --- | --- | --- | --- | --- |
| *if* ***YES****, go to question 4*  *if* ***NO****, go to question 2* | | | | |  |
| 1. **Is there a reason why you haven’t spoken to anyone else about living with kidney disease?** *(circle all that apply)*   Not been offered to me Not interested in talking to others  Have enough support around me Other(s) *(please state below)*  ………………………………………………………………………………………………………………………………………………. | | | | |  |
| 1. **If peer support was offered to you, would you like to speak to someone?** *(circle your answer)*   YES NO NOT SURE  **PLEASE GO TO QUESTION 11** | | | | |  |
| If you answered **‘YES’** to question 1   1. **Who did you speak to?**   *(Mark an [X] in all boxes that apply)*  Family member  Spouse  Person with chronic kidney disease that was unknown to me  Friend  A group of people  Other *(please state)*………………………………………………………………………………………………………………… | | | | |  |
| 1. **Where did this encounter take place?** (*circle all that apply)*   At home In hospital Online At a community event  Via a patient charity Via a peer support program  Somewhere else *(please state)*………………………………………………………………………………………………. | | | | |  |
| 1. **In the last 6 months, approximately how many times have you spoken to someone else that is not part of your medical team, about your kidney disease?**   ……………………………………………………………………………………………………………………………………………… | | | | |  |
| 1. **What topics did you discuss?**   *(Mark an [X] in all boxes that apply)*  The experience of living with kidney disease  Making decisions about treatment options  Psychological aspects of living with kidney disease e.g., worry, depression  Coping with living with chronic kidney disease  Diagnosis of kidney disease  Other health conditions  Carrying out usual activities whilst living with kidney disease  Managing life on dialysis/after transplantation  Anything else *(please state)* …………………………………………………………………………………………… | | | | |  |
| 1. **I found talking to another person about my kidney disease to be:**   *(Mark an [X] in one box)*  not at all useful quite useful useful very useful | | | | |  |
| 1. If you found it ***quite useful/useful/very useful*** talking to another person, **please say why.**   It helped me to:  *(Mark an [X] in all boxes that apply)*  Cope better with living with my illness  Make a decision about which treatment to choose  Manage my daily living tasks  Adjust to my diagnosis  Understand what it is like to have dialysis/live with a transplant  Answered questions I/my healthcare team did not know the answer to  Talk socially with someone in the same situation, and not have to explain my feelings  Prepare to talk with family/friends  Prepare to talk with health professionals  Anything else *(please state*)………………………………………………………………………………………………… | | | | |  |
| 1. If you ***did not*** ***find it useful*** talking to another person, **please say why** *[otherwise move on to question 11]:*   *(Mark an [X] in all boxes that apply)*  The experience made me feel worried  I didn’t think the person’s experiences were like mine  I found it difficult to have an honest and open discussion  I didn’t think I had anything in common with the person I spoke to  The person had a limited experience of the different treatment options  I have enough support from friends and family  I don’t want to spend more time with people who are more ill than I am  I prefer to get my health information from my health professional team  I want to spend more time on other activities in my life  *Question continues on the next page….*  I prefer to think through the issues on my own, and with my family  Raised questions that I hadn’t thought about before  Anything else *(please state*)……………………………………………………………………………………………………….  ………………………………………………………………………………………………………………………………………………….. | | | | |  |
| 1. **It was important to me that the person I spoke to was….**   …a similar age to me | | | | |  |
| Not at all important | Quite important | Important | | Very important |  |
| ...the same sex as me | | | | |  |
| Not at all important | Quite important | Important | | Very important |  |
| …had the same ethnic and/or religious background as me | | | | |  |
| Not at all important | Quite important | Important | | Very important |  |
| …from the same hospital | | | | |  |
| Not at all important | Quite important | Important | | Very important |  |
| 1. **Have you provided peer support to another person?** | | | YES NO    *if* ***YES****, go to question 12*  *if* ***NO****, go to question 15* | |  |
| 1. **In the last 6 months, as a peer supporter, approximately how many times have you spoken to someone else about your kidney disease?**   ……………………………………………………………………………………………………………………………………………… | | | | |  |
| 1. **Who did you speak to?**   *(Mark an [X] in all boxes that apply)*  Family member  Spouse  Person with chronic kidney disease that was unknown to me  Friend  A group of people  Other(s) *(please state)*……………………………………………………………………………………………………………… | | | | |  |
| 1. **Where did this encounter take place?** (circle *all that apply)*   At home In hospital Online At a community event  Via a patient charity Via a peer support program  Somewhere else *(please state)*……………………………………………………………………………………………. | | | | |  |
| 1. **There are people in my life who….** *(Mark an [X] in one box on each line)* | | | | |  |
| ….pay attention to my feelings | | |  | |  |
| *Strongly disagree Disagree Neutral Agree Strongly Agree* | | | | |  |
| ….appreciate what I do | | |  | |  |
| *Strongly disagree Disagree Neutral Agree Strongly Agree* | | | | |  |
| ….I can get help from if I need it | | |  | |  |
| *Strongly disagree Disagree Neutral Agree Strongly Agree* | | | | |  |
| ….I can talk to about how to handle things | | |  | |  |
| *Strongly disagree Disagree Neutral Agree Strongly Agree* | | | | |  |

**Section 2: Decision making and expectations about treatment**

| This section asks your views and experience of dialysis and/or transplant.   1. **At this time, which answer best describes the stage of your treatment decision?**   *(Mark an [X] in one box from the following six options)* | |
| --- | --- |
| I haven’t begun to think about the choices |  |
| I haven’t begun to think about the choices but am interested in doing so |  |
| I am considering the options now |  |
| I am close to selecting an option |  |
| I have already made a decision; but am still willing to reconsider |  |
| I have already made a decision and am unlikely to change my mind |  |
| This next set of questions asks you to consider how certain you feel about your decision.  *(circle one option on each line)* | |
| 1. **Did you feel sure about the best choice for you?** | YES NO |
| 1. **Did you know the benefits and risks of each option?** | YES NO |
| 1. **Were you clear about which benefits and risks matter most to you?** | YES NO |
| 1. **Did you have enough support and advice to make a choice?** | YES NO |
| 1. **I find that dialysis/transplantation…**   *(Mark an [X] in one box on each line)* | |
| .…helps me to cope with my symptoms |  |
| Partially disagree Partially agree Agree Definitely agree | |
| ….makes my symptoms disappear |  |
| Partially disagree Partially agree Agree Definitely agree  *Question continues on the next page…* | |
| ….improves my energy |  |
| Partially disagree Partially agree Agree Definitely agree | |
| …. improves my day to day living | |
| Partially disagree Partially agree Agree Definitely agree | |
| …makes my symptoms considerably better |  |
| Partially disagree Partially agree Agree Definitely agree | |
|  | |
|  | |
|  | |
|  | |
|  | |
|  | |
|  | |
|  | |
|  | |
|  | |
|  | |
|  | |
|  | |
|  | |
|  | |
|  | |
|  | |
|  | |
|  | |
|  | |
|  | |
|  | |

**Section 3: Your Health**

This section asks you questions about your health and your quality of life. For each question, mark an [X] in one box on each line.

| 1. **In general, would you say your health is:**   Poor Fair Good Very good Excellent | | | | | | | | | | | | | | | | | |
| --- | --- | --- | --- | --- | --- | --- | --- | --- | --- | --- | --- | --- | --- | --- | --- | --- | --- |
| 1. The following items are about activities you might do during a typical day. **Does your health now limit you in these activities? If so, how much?** | | | | | | | | | | | | | | | | | |
|  | | Yes, limited a lot | | | | | | | Yes, limited a little | | | | | No, not limited at all | | | |
| Moderate activities, such as moving a table, pushing a vacuum cleaner. | |  | | | | | | | | | | | | | | | |
| Climbing several flights of stairs | |  | | | | | | | | | | | | | | | |
| 1. **During the past 4 weeks, have you had any of the following problems with your work or other regular daily activities as a result of your physical health?** | | | | | | | | | | | | | | | | | |
|  | | Yes No | | | | | | | | | | | | | | | |
| Accomplished less than you would like | |  | | | | | | | | | | | | | | | |
| Were limited in the kind of work or other activities | |  | | | | | | | | | | | | | | | |
| 1. **During the past 4 weeks, have you had any of the following problems with your work or other regular daily activities as a result of any emotional problems (such as feeling depressed or anxious)?** | | | | | | | | | | | | | | | | | |
|  | | Yes No | | | | | | | | | | | | | | | |
| Accomplished less than you would like | |  | | | | | | | | | | | | | | | |
| Didn’t do work or other activities as carefully as usual | |  | | | | | | | | | | | | | | | |
| 1. **During the past 4 weeks, how much did pain interfere with your normal work (including both work outside the home and housework)?** | | | | | | | | | | | | | | | | | |
| Not at all A little bit Moderately Quite a bit Extremely | | | | | | | | | | | | | | | | | |
| 1. These questions are about how you feel and how things have been with you during the past 4 weeks. For each question, please give the one answer that comes closest to the way you have been feeling. **How much of the time during the past 4 weeks…** | | | | | | | | | | | | | | | | | |
|  | | None of the time | | | A little of the time | | | | Some of the time | A good bit of the time | | | | Most of the time | | | All of the time |
| Have you felt calm and peaceful? | |  | | | | | | | | | | | | | | | |
| Did you have a lot of energy? | |  | | | | | | | | | | | | | | | |
| Have you felt unhappy? | |  | | | | | | | | | | | | | | | |
| 1. During the past 4 weeks, **how much of the time has your physical health or emotional problems interfered with your social activities (like visiting with friends, relatives, etc.)?** | | | | | | | | | | | | | | | | | |
| None of the time | A little of the time | | | Some of the time | | | | Most of the time | | | | | All of the time | | | | |
|  | | | | | | | | | | | | | | | | | |
| **Your Kidney Disease** | | | | | | | | | | | | | | | | | |
| 1. How **true or false** is each of the statements to you? | | | | | | | | | | | | | | | | | |
|  | Definitely false | Mostly false | | | | Don’t know | | | | | Mostly true | | | | Definitely true | | |
| My kidney disease interferes too much with my life |  | | | | | | | | | | | | | | | | |
| Too much of my time is spent dealing with my kidney disease |  | | | | | | | | | | | | | | | | |
| I feel frustrated dealing with my kidney disease |  | | | | | | | | | | | | | | | | |
| I feel like a burden on my family |  | | | | | | | | | | | | | | | | |
| I have problems with my catheter *(peritoneal dialysis patients only)* |  | | | | | | | | | | | | | | | | |
| I have problems with my catheter site *(Haemodialysis patients only)* |  | | | | | | | | | | | | | | | | |
| 1. **During the past 4 weeks, to what extent were you bothered by each of the following?** | | | | | | | | | | | | | | | | | |
|  | Not at all bothered | | Somewhat bothered | | | | Moderately bothered | | | | | Very much bothered | | | | Extremely bothered | |
| Soreness in your muscles |  | | | | | | | | | | | | | | | | |
| Chest pain |  | | | | | | | | | | | | | | | | |
| Cramps |  | | | | | | | | | | | | | | | | |
| Itchy skin |  | | | | | | | | | | | | | | | | |
| Dry skin |  | | | | | | | | | | | | | | | | |
| Shortness of breath |  | | | | | | | | | | | | | | | | |
| Faintness or dizziness |  | | | | | | | | | | | | | | | | |
| Lack of appetite |  | | | | | | | | | | | | | | | | |
| Washed out or drained |  | | | | | | | | | | | | | | | | |
| Numbness in hands and feet |  | | | | | | | | | | | | | | | | |
| Nausea or upset stomach |  | | | | | | | | | | | | | | | | |

**Effects of Kidney disease on your daily life**

| 1. Some people are bothered by the effects of kidney disease on their daily life, while others are not. **How much does kidney disease bother you in each of the following areas?** | | | | | |
| --- | --- | --- | --- | --- | --- |
|  | Not at all bothered | Somewhat bothered | Moderately bothered | Very much bothered | Extremely bothered |
| Fluid restriction |  | | | | |
| Dietary restriction |  | | | | |
| Your ability to work around the house |  | | | | |
| Your ability to travel |  | | | | |
| Being dependent on doctors and other medical staff |  | | | | |
| Stress or worries caused by kidney disease |  | | | | |
| Your sex life |  | | | | |
| Your personal appearance |  | | | | |
|  |  | | | | |

**Section 4: Managing and coping with chronic kidney disease treatments**

We are interested in finding out about the effort you have to make to look after your health and how this impacts on your day-to-day life.

Please tell us how much ***difficulty*** you have with the following: *(Mark an [X] in one box on each line)*

|  | Not difficult | A little | Quite | Very | Extremely | Does not apply |
| --- | --- | --- | --- | --- | --- | --- |
| 1. Taking lots of medications |  | | | | | |
| 1. Remembering how and when to take medication |  | | | | | |
| 1. Paying for prescriptions, over the counter medication or equipment |  | | | | | |
| 1. Collecting prescription medication |  | | | | | |
| 1. Monitoring your medical conditions (e.g. checking your blood pressure, blood sugar etc.,) |  | | | | | |
| 1. Arranging appointments with health professionals |  | | | | | |
| 1. Seeing lots of different health professionals |  | | | | | |
| 1. Attending appointments with health professionals (e.g. time off work etc.,) |  | | | | | |
| 1. Getting health care in the evenings and at weekends |  | | | | | |
| 1. Getting help from community services (e.g. district nurses, social worker etc.) |  | | | | | |
| 1. Obtaining clear and up-to-date information about your condition |  | | | | | |
| 1. Making recommended lifestyle changes (e.g. diet and exercise etc.) |  | | | | | |
| 1. Having to rely on help from family and friends |  | | | | | |

**Section 5: Personal Details**

These final questions ask you some personal details. Your answers are treated in confidence.

| How old are you? …………………… years | | | | | | | | | | | |
| --- | --- | --- | --- | --- | --- | --- | --- | --- | --- | --- | --- |
| What is your sex? *(circle your answer)*  Female Male Non- binary Prefer not to say | | | | | | | | | | | |
| What is the highest level of education you have received? *(Mark an [X] in one box)* | | | | | | | | | | | |
| Professional qualification | | | | | | | | | |  | |
| ‘O’ level / GSCEs | | | | | | | | | |  | |
| Apprenticeship and/or NVQ (National Vocational Qualification) | | | | | | | | | |  | |
| ‘A’ level/‘Highers’ | | | | | | | | | |  | |
| Degree or higher | | | | | | | | | |  | |
| How you would describe your ethnic origin? *(circle one option)* | | | | | | | | | | | |
| White | Indian | | | Pakistani | | Bangladeshi | | | Chinese | | Black-Caribbean |
| Black-African | Black-British | | | Other …………………………………………………………………………………. | | | | | | | |
| Which of the following best describes your current work status? *(Mark an [X] in one box)* | | | | | | | | | | | |
| Working full-time (for 30 hours or more per week) | | | | | | | | | |  | |
| Working part-time (for less than 30 hours per week) | | | | | | | | | |  | |
| Unemployed due to illness or disability | | | | | | | | | |  | |
| Unemployed for another reason | | | | | | | | | |  | |
| Retired due to illness or disability | | | | | | | | | |  | |
| Retired for another reason | | | | | | | | | |  | |
| Home maker (housewife/househusband) | | | | | | | | | |  | |
| Other ……………………………………………………………………………………………………… | | | | | | | | | |  | |
| Has your work status changed because of your kidney problem?  *(circle one answer)* | | | | | | | | | | YES NO | |
| What is your marital status? (*circle one answer)* | | | | | | | | | |  | |
| Married | | Living with a companion | | | Single | | | Widowed | | Divorced | |
| Who else lives with you, in your home? *(circle ALL that apply)* | | | | | | | | | |  | |
| Children | | | Parents | | | | Other relatives | | | Friends | |
| Other……………………………………………………………………………………………………….. | | | | | | | | | |  | |
| What is your yearly household income? *(circle one answer)* | | | | | | | | | |  | |
| £20,000 or less | | | | | £21,000 - £40,000 | | | | | £41,000-60,000 | |
| £61,000-£80,000 | | | | | £81,000-100,000 | | | | | £101,000 + | |
|  | | | | |  | | | | |  | |

|  | On my own | With help from a friend or relative | With help from a member of staff | |
| --- | --- | --- | --- | --- |
| 1. How did you complete this questionnaire? |  |  | |  |

**Thank you** for sharing your experiences with our team so we can find out what helps people manage their kidney disease, and when it may be useful to let others know about peer support for chronic kidney disease. You will receive a second and final questionnaire in about 6 months.

***Please return the questionnaire in the stamped addressed envelope provided***

If you have any concerns, please speak to your kidney healthcare professional team in the first instance or contact Dr Anna Winterbottom who is leading this project, [anna@winterbottom.co.uk](mailto:anna@winterbottom.co.uk), telephone: 07981 689 434.
