# Supplementary material for: The role of peer support in coping and adjustment to dialysis and transplantation: Study protocol
Source: PLoS One. 2025 Feb 10;20(2):e0318124. doi: 10.1371/journal.pone.0318124 (PMC11809911; doi:10.1371/journal.pone.0318124)
Supplement: S3 File — Pre-treatment interview schedule, Time 1. (DOCX) [file pone.0318124.s003.docx]

**Introduction**

Thank you for agreeing to speak to me. I would like to speak to you today about your experience of having chronic kidney disease. Chronic kidney disease means that your kidneys have been damaged and they no longer work properly.

You will have read the invitation letter and patient information sheet which explains that we want to talk to you about your experience of talking to other people with chronic kidney disease about your illness, this is known as ‘peer support’.

People use the term ‘peer support’ in different ways. Our study team describes peer support as:

… when people with similar long-term health conditions or health experiences come together to support each other to cope with their illness.

Peer support can happen formally when organised by a health service or charity, and informally when talking with a relative or another patient or family member. It can be part of a one-to-one conversation or as part of a group, in face-to-face or on-line communications. Peer support can happen when you are at home, at hospital, in community settings or travelling.

We are interested in people’s different experiences of peer support, including people who *haven’t experienced* any at all.

I will also ask you some questions about what it is like to have kidney disease, and what you expect life to be like when you start dialysis/receive a transplant.

If there are any questions that I ask you that make you feel uncomfortable and you don’t wish to answer, please let me know and we will move on to the next one. You can also stop the interview at any point if you don’t wish to continue, just let me know. This will not affect your treatment at the kidney unit.

Before we begin, do you have any questions about our conversation today or the project?

***Peer support, information and expectations of treatment***

1. Do you know anyone else with kidney disease?

*Probe: What relation are they to you? Family/friends/in clinic/via formal peer support/pre-dialysis workshop*

1. Have you spoken to them/anyone else with kidney disease about your illness?
2. Have you discussed with anyone else how you will manage treatment?

*Prompt: how did the conversation come about? how many times talked to them/location of conversation*

1. What did you discuss?

**If no, would you like to?** What would you like to discuss?

1. Did talking to someone else provide you with additional information to that which you received from the hospital?
2. Did talking to someone else help? If so, how/how not?

*Prompt: to make treatment decision, cope and adjust, reassurance.* ***Important to probe if NOT useful, and why.***

1. How has peer support helped you?

*Prompt: help make a decision, helped to adjust/cope, discussion about end of life, discussion about symptoms, something else?*

1. What do you think are the positive and negative aspects of peer support?
2. Do you think that it is appropriate for hospitals to provide peer support or should some other organisation e.g., a charity, do this?
3. Do you think that doctors and nurses understand what it is like to have kidney disease?

*Prompt: How could this be improved/what do they understand well?*

1. Would regular feedback on the patient experience help doctors and nurse provide better care?

*Prompt: can you see any difficulty with providing this type of feedback?*

***Patients understanding of illness, coping and adjustment***

1. What were your first thoughts and feelings about having kidney disease?

*(prompts: how changed? Better/ worse)*

1. Can you tell me about how you were diagnosed with kidney disease?

*(prompts: cause, Identity, timeline, consequences, control)*

1. What do you think caused your kidney disease?
2. What other conditions do you have to live with/manage alongside your kidney problem?
3. How important are these compared to your kidney problem?
4. How do you feel now about your diagnosis? Has this feeling changed from when you were first diagnosed? Why? Acceptance? Better treatment? Life expectancy?
5. How does your kidney disease impact on your everyday life?

*Prompt: what activities do you enjoy taking part in? does your KD impact on the enjoyment of these?*

1. How do you think it will impact on how you live in the future?
2. Do you have anyone that helps support you with everyday living and managing your illness?
3. What medication do you take? Do you manage to take it regularly? If not why not?
4. Do you have side effects from the medicine?
5. Does medication stop you from doing things you would normally do?
6. Is it easy/difficult do you find it to make and keep medical appointments?
7. How frequently do you travel to hospital or GP or other?
8. keep track of medical appointments with different healthcare provider
9. Have you experienced difficulties with any of the following:

- problems with different health care providers not communicating with each other about my medical care

- Having to see too many different specialists for my health problem(s) or illness(es)

- problems filling out forms related to my health care

- problems getting appointments at times that are convenient for you

- problems getting appointments with a specialist

- waiting too long at my medical appointments

- waiting too long at the pharmacy for my medicine

1. How long do appointments with your doctor usually last?
2. Where do they occur?

*Prompt: telephone, face to face, online. Are they long enough to discuss your concerns?*

1. What sort of things do you like to do in your leisure time? (hobbies, travel etc)
2. Does CKD/treatment affect your social life and/or leisure activities? If yes, how does this make you feel?
3. Are you encouraged to keep track of your health behaviours, e.g., tracking exercise, fluid/foods you eat, or medicines you take?

- How successful are you in managing to do this?

1. Are you encouraged to monitor your health condition, e.g., weighing yourself, checking blood pressure, or checking blood sugar?

- How successful are you in managing to do this?

1. Does having CKD/treatment prevent you from doing anything you wish to do? How does this make you feel?

*Prompt: socialising, shopping, washing dressing, cooking, enjoying hobbies, religious activities etc.,*

1. What has been helpful to you in the way you manage and live with CKD?
2. What has been unhelpful to you in the way you manage and live with CKD?
3. What are your expectations how you will manage your illness in the future?
4. Do you feel able to discuss the future of your treatment with healthcare staff and/or family?

***Decision making process***

1. Can you tell me about any treatments you are considering to manage your kidney disease.

*Prompt: Tell me how you were informed about the options dialysis and Tx? (nurse, doctor, friend, carer, patient)*

1. Do you get a sense that some health professionals favour one form of treatment over another? What makes you think that?
2. Do you think that some doctors/nurses favour one form of treatment over another? What makes you think that?
3. Do you think there are some reasons why some patients should not be offered both types of treatment? Why do you think that?
4. Did you do anything that helped you make sense of the information to you and how you wanted to live your life?
5. Is there any information about your disease or treatment options that you would like to receive that you have not received?
6. What information was helpful to you? (unhelpful – with hindsight was there anything misleading about the information you were given?)
7. Have you done any research to understand your kidney disease and the treatment options better? Did you find this information useful to you?
8. If you were to offer advice to someone else about how to think about choosing between dialysis and/or transplant, what would you say?
9. If you were going to offer advice on how someone else should think about the advantages and disadvantages of dialysis and/or transplant, what would you say?
10. How do you think that treatment will impact on your life when it starts (post dialysis/post transplant)?

*Prompt: feel physically better/be able to do more enjoyable things, reduce symptoms/lengthen life etc.*

Thank you, I have now asked you all of the questions are there any issues/questions that you would like to talk about that have not been covered? How do you feel at the end of our conversation? If you would like to talk to someone about the topics we discussed today then do get in touch with your kidney care team or the National Kidney Federation/Kidney Care UK helpline.

Once again that everything you have told me today is confidential and completely anonymous. If you would like to be kept in touch about the results I can contact you when they are available. If yes, what is the best way to contact you?
